# Application of Optical Coherence Tomography (OCT) for Diagnosis of Caries, Cracks, and Defects of Restorations

**DOI:** 10.1007/s40496-015-0045-z

**Published:** 2015-04-09

**Authors:** Yasushi Shimada, Alireza Sadr, Yasunori Sumi, Junji Tagami

**Affiliations:** Department of Cariology and Operative Dentistry, Graduate School of Medical and Dental Sciences, Tokyo Medical and Dental University, Tokyo, Japan; International Exchange Center, Tokyo Medical and Dental University, Tokyo, Japan; Division of Oral and Dental Surgery, Department of Advanced Medicine, National Hospital for Geriatric Medicine, National Center for Geriatrics and Gerontology, Obu, Aichi Japan

**Keywords:** Optical coherence tomography (OCT), Caries, Tooth fracture, Interfacial gap, Diagnosis

## Abstract

Optical coherence tomography (OCT) is a noninvasive technique providing cross-sectional images of a tooth structure. This review describes the use of OCT for detecting dental caries, tooth fractures, and interfacial gaps in intraoral restorations. OCT can be a reliable and an accurate method and a safer alternative to X-ray radiography.

## Introduction

Optical coherence tomography (OCT) is an emerging diagnostic method for cross-sectional imaging of internal biological structures. OCT helps visualize differences in tissue optical properties, which includes the effects of both optical absorption and scattering. It is actually an interferometric technique which uses infrared light waves that reflect off the internal microstructure in a way that, in principle, is analogous to an ultrasonic pulse echo [1, 2].

The first in vitro and in vivo images of dental hard and soft tissue with OCT were acquired by Colston et al. in 1998 [3, 4–6]. The early OCT systems were based on time-domain (TD) detection in which echo time delays of light were identified by measuring the interference signal as a function of time, while scanning the optical path length of the reference arm [1, 2]. OCT combines light from a low-coherence light source with a Michelson interferometer to produce cross-sectional images of tissue structures generated as a result of interaction between a partially coherent beam of optical radiation and tissue components.

Cross-sectional images are generated by multiple axial measurements of echo time delay (axial scans or A-scans) and scanning of the incident optical beam transversely. This generates a two-dimensional data set, which represents the optical backscattering in a cross-sectional plane through the tissue. Images or B-scans can be displayed in a false color or gray scale to visualize tissue changes [1, 2]. Acquiring serial cross-sectional images by scanning the incident light beam in a raster pattern can produce three-dimensional volumetric data sets [2].

Dental caries is an infectious microbial disease that results in localized dissolution and destruction of calcified dental tissues [7, 8]. Despite considerable decline in the incidence of caries, the disease is yet to be completely eradicated, particularly in children and young adults. The current international trend in caries management is moving away from the surgical model toward a preventive approach, which aims to control the initiation and progression of the disease [9]. Therefore, dentists need an imaging technique that can noninvasively and reliably quantify the extent of caries. Large noncavitated dentin lesions have been frequently observed beneath seemingly sound occlusal caries in children, with a prevalence of around 15 % and a possible high of 50 % [10]. Regular use of fluorides reinforces and remineralizes the superficial enamel, which may obscure underlying lesions in dentin (so-called “hidden caries”) [11].

In general, there are different types of clinical scenarios wherein OCT could have important applications.

1. Dental X-rays considerably underestimate caries lesion size and are not sensitive enough. By the time a lesion is visible on radiographs, the demineralization has extended to or beyond the middle third of the dentin [12, 13••, 14].
2. Tooth cracks have been a diagnostic challenge because of the difficulty in locating the fracture lines of an incomplete fracture [15, 16, 17••]. Early detection and diagnosis are important to limit crack growth [15, 16].
3. Clinical assessments of margin quality for intraoral restorations are routinely carried out in dental practice; however, the replacement of existing restorations and the decisions related to treatment planning are very subjective [18–20].

The application of OCT in dentistry for imaging teeth and composite restorations may facilitate clinical diagnosis of caries and detection of restorative failures in the future [13••, 17••, 18, 21, 22]. This review paper discusses the development of a dental OCT system and its application for diagnosis of dental caries and tooth fractures as well as detection of interfacial gaps of adhesive restorations.

## OCT Systems in Dentistry and Caries Diagnosis

OCT has been revolutionized in recent years by the development of Fourier-domain (FD) techniques that provide a distinct increase in sensitivity compared with traditional (TD)-OCT [23]. Swept-source (SS)-OCT is one of the implements of FD-OCT and employs a wavelength-tuned laser as the light source [24].

In SS-OCT, the spectrally resolved interference is derived from rapidly sweeping the wavelength of the laser [2, 24]. The axial resolution of SS-OCT is ultimately set by the linewidth of the laser beam [25]. The transverse resolution is determined by the focus spot size on the sample [25]. The high acquisition speed of SS-OCT, providing near real-time video-rate imaging while improving the overall signal-to-noise ratio of the acquired images, has made clinical applications of OCT more feasible [26].

Several lines of evidence indicate that the infrared region from 780 to 1550 nm can offer great potential for optical imaging of enamel [3–6] because of weak scattering and absorption in this region, especially around 1310 nm [27, 28]. At longer wavelengths, water absorption increases significantly and reduces the penetration of near-infrared light [2]. In SS-OCT, sound enamel is almost transparent at the SS-OCT wavelength range or the upper near-infrared region around 1300 nm [13••, 17••, 21, 27, 28]. Since the optical characteristics of the enamel and dentin differ because of structural and compositional factors, the two structures can be distinguished from each other with the dentin–enamel junction (DEJ) appearing as a dark borderline [13••, 17••, 21, 29].

In the case of sound dentin, the measurable signal depth will be relatively smaller because of higher attenuation [13••, 17••, 22, 30]. The orientation of the OCT beam in relation to the inclination of the structure can affect the signal intensity, i.e., a steep slope on the enamel surface may not appear as bright as a less steep surface [31]. Moreover, signal intensity and attenuation patterns are affected by caries [13••, 21, 22, 29, 32, 33•, 34]. Therefore, the effective imaging depth of OCT on a tooth depends on the structure being imaged; this depth has been considered to be 2–3 mm in many cases.

It is well known that carious lesions can possess an intact surface zone that can appear similar to that of sound enamel [35]. A hydroxyapatite crystal is known to be birefringent, and the de- and remineralization processes associated with carious lesions are known to alter birefringence [36]. If the sample is birefringent with an anisotropic refractive index, it modifies the polarization state of light passing through it [27, 28, 37–40].

Polarization-sensitive (PS)-OCT was developed in 1992 using a pair of detectors to record the two orthogonal polarization states of light backscattered from tissue and to measure its birefringence [37]. Using polarization-maintaining fiber in the system, a detector can measure the signal parallel to the incident polarization axis, and another detector can measure the signal in the perpendicular or cross-polarization axis [38]. Polarized light can be described by six degenerate polarization states of light, four linear and two circular, used to construct the Mueller matrix-Stokes vector formalism, and by an elliptical state [39]. Mueller matrix elements in dental hard tissue were analyzed to observe the differences between sound and demineralized enamel [39].

Fried et al. reported that PS-OCT can detect and quantify surface demineralization by using linearly polarized incident light and measuring the backscattered signal in two orthogonal axes [40]. It was observed that the unfavorable strong reflections from the tooth surface were greatly reduced in the orthogonal polarization image in PS-OCT, enabling better resolution of the surface zone of the lesion [40]. Therefore, PS-OCT has been successfully used to image incipient lesions in order to quantify lesion depth and mineral loss in the enamel or dentin [38–50]. Moreover, attempts have been made to use the polarization memory effect in PS-OCT for assessment of early dentin demineralization [50].

**Fig. 1:**
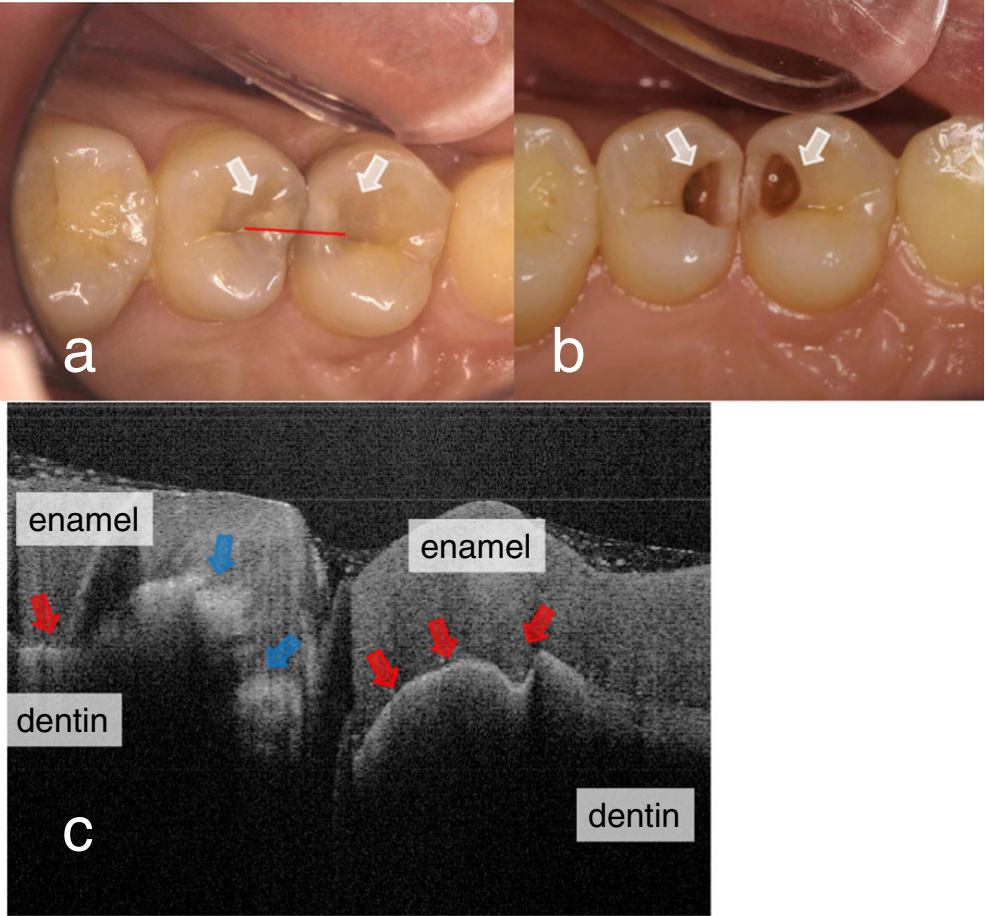
Dental caries in first and second premolars. **a** Occlusal view before the surgical treatment. Underlying dark shadows were visually observed at the first and second premolars (*arrow*). SS-OCT observation was performed along *red line*. **b** Occlusal view during the cavity preparation. Presence of deep lesions with softened dentin was obvious (*white arrow*). **c** SS-OCT image at *red line* in (**a**) before cavity preparation. *Bright zone* indicates the increased light scattering in porous demineralized tissue (*blue arrow*). A strong reflection penetrating along the DEJ indicates the lesion is “cavitated” (*red arrow*)

**Fig. 2:**
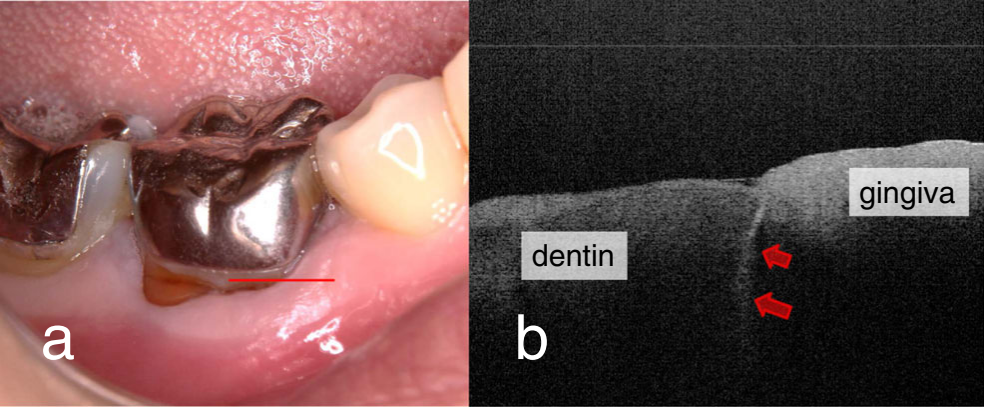
Mandibular first molar with occlusal pain. **a** Buccal view. Full coverage metal crown complicated the visual inspection and clinical diagnosis. SS-OCT observation was performed along the *red line* in order to image the cross-sectional view of cervical zone horizontally. **b** SS-OCT image at *red line* in (**a**). A strong reflection penetrating into dentin indicates presence of vertical crack in root dentin (*red arrow*)

**Fig. 3:**
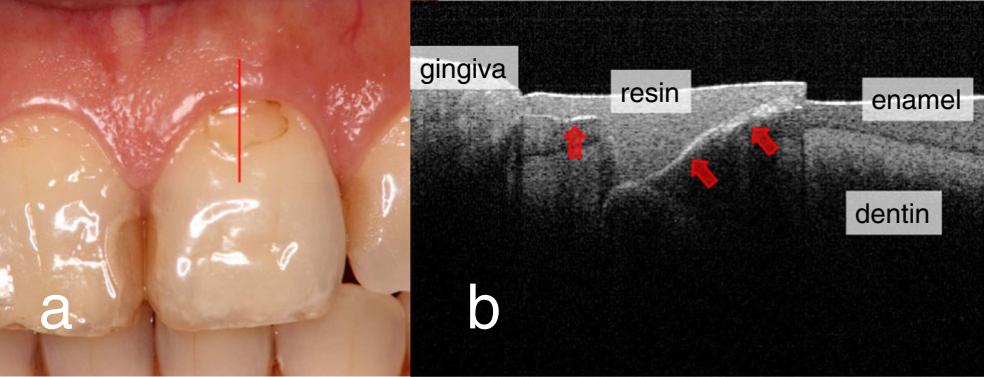
Clinical assessment of resin composite restoration. **a** Buccal view. Class V composite restoration was present with slight marginal discoloration but without any clinical symptoms. SS-OCT observation was performed along the *red line*. **b** SS-OCT image at *red line* in (**a**). Distinct white border between resin and enamel/dentin indicates “gap” (*red arrow*)

Strong reflection from the material surfaces appears to be effective in conventional OCT setups for detecting the reflective signal from the fault, e.g., the border of cavitated caries, tooth fractures, and interfacial gaps of tooth restorations [13••, 17••, 18–20, Figs. 1, 2, and 3]. In addition, several lines of evidence report that the reflectivity of smooth surface caries lesions can be distinguished against sound enamel with OCT without polarization sensitivity, and the lesion contrast can be evaluated by post acquisition image processing [22, 29, 30, 32, 33•, 51–54].

In OCT, tooth demineralization can be distinguished from sound tissue based on the following two main principles: increased light scattering in porous demineralized tissue [22, 29, 32, 33•, 34, 51–54] and depolarization of incident light by demineralized tissue [27, 28, 38–50]. The latter necessitates PS-OCT or cross-polarization OCT in which the former phenomenon can be observed by both conventional and PS-OCT setups as increased signal intensity [22, 29, 32, 33•, 34, 51–54].

Our previous studies have shown that SS-OCT without polarization sensitivity is capable of imaging the cavitated hidden lesions beneath seemingly sound caries both in vitro and in vivo [13••, 21, 55]. In the case of caries with extensive loss of enamel and dentin creating a hollow space, the upper borders of the hollow space strongly scatter and attenuate light, which is clearly imaged in SS-OCT without polarization sensitivity. We confirmed that the high imaging resolution and penetration depth of SS-OCT is advantageous in some clinical cases, especially for detection of cavitated caries or deeper lesions [13••, 21, 55•]. On the other hand, Lenton et al. tried to use cross-polarization swept-source OCT (CP-OCT) to assess the presence of secondary caries at subsurface composite restorations [56]. Further investigation is required regarding a comprehensive comparison of the clinical applications of the two OCT approaches in dentistry.

Once the caries has progressed, it exhibits only low polarization change because a huge loss of mineral content can reduce birefringence as well as scattering [38]. When the caries progresses and results in cavity formation, the signal intensity in the margin of the defect increases in SS-OCT, resulting in appearance of bright cavity borders that can readily be distinguished from a noncavitated demineralized lesion [13••, 21, 55]. A similar optical phenomenon has been reported as the basis for detection of defects under dental restorations [19, 20].

In enamel caries, the signal generally increases and the demineralized region appears brighter on grayscale OCT images [13••, 21, 29, 33•]. There is a 2–3 order of magnitude increase in the scattering coefficient of dental enamel on demineralization [57]. In general, enamel demineralization results in an increase in porosity because of mineral loss in the body of the lesion [58••]. It is well known that the reflection of light occurs between two homogeneous media having different refractive indices. It is highly probable that micro interfaces between demineralized mineral crystals and water within the pores cause higher reflectivity, thus resulting in increased brightness in the corresponding OCT image [58••]. Furthermore, the formation of pores on a size scale similar to the wavelength of the light may act as Mie scatterers [59]. A study using Monte Carlo simulations for imaging caries lesions suggests that the optimal spectral region for the highest lesion contrast depends on the lesion depth and severity. The shorter wavelengths may yield higher contrast for shallow lesions while longer wavelengths should yield higher contrast for deeper lesions [60].

Jones et al. reported that the reflectivity of demineralized enamel can be measured nondestructively and that the integrated reflectivity correlates with the mineral loss [41, 42]. Mineral volume (MV) changes arising from remineralization can decrease the optical reflectivity. However, enamel demineralization of 30 and 70 % MV do not have significantly different scattering intensities even in PS-OCT [42].

Refractive index (*n*) is an important parameter in light propagation through biological tissues, including teeth. The *n* can serve as an indicator of the scattering properties of tissue, as scattering itself is the end result of local *n* variation [61]. Hariri et al. measured *n* of de- and remineralized enamel and dentin using SS-OCT without polarization sensitivity and mineral content using transverse microradiography [58••]. As a result, de- and remineralization of enamel and dentin showed measurable changes in *n* in SS-OCT [58••]. The *n* and MV obtained ranged from 1.52 to 1.63 and 50 to 87 (vol %) in enamel and from 1.43 to 1.57 and 11 to 48 (vol. %) in dentin, respectively [58••].

In the case of dentin caries, SS-OCT can image the continuous bright area through the enamel into the dentin [21, 29]. The depth of demineralization and caries penetration into the dentin can be estimated by considering the location of the DEJ as a reference point (Fig. 1). If the caries penetrates into the dentin, the penetration depth of the bright zone observed in SS-OCT extends beyond the DEJ level [13••, 21, 29]. In some cases, lateral expansion of the caries lesion creates microgaps along the DEJ, where strong reflection occurs. Increased signal intensity along the DEJ adjacent to the caries indicates involvement of this junction, accompanied by structural disintegration of the enamel and dentin [13••, Fig. 1]. However, incident light of OCT undergoes significant attenuation, which hampers the process of obtaining sufficient signal intensity [13••, 21, 29, 30].

Amaechi et al. measured the percent reflectivity loss due to demineralization on dentin surfaces and showed that this correlated well with the mineral loss from microradiography [30]. In dentin caries, a hydrated organic matrix, such as micro-debris and collagen, is left at the lesion because of demineralization [13••, 21, 29, 31]. Collagen is optically nonlinear and is known to scatter light [62].

The attenuation coefficient of an OCT signal can be an objective quantitative parameter for distinguishing sound from carious enamel and dentin [33•, 53]. The 850-nm OCT signal has been shown to attenuate lesser in carious enamel than in sound enamel [53]. OCT signal attenuation in a demineralized tooth appears to be influenced by the wavelength of the incident light. A 1310-nm OCT signal exhibits a higher attenuation coefficient in demineralized enamel and dentin over that of sound tissues [33•, 63], while attenuation of a 1310-nm OCT signal could also be adopted as a clinical parameter for detection of demineralization [64].

In our study, most SS-OCT images obtained from the occlusal surfaces could not clearly display the pulp chamber within the tooth, and it was impossible to observe the distance of the caries from the dental pulp [13••, 21]. Consequently, radiographic examination is still considered to be necessary in cases exhibiting significant irreversible pulpitis symptoms. On the other hand, SS-OCT can display the location of the pulp beneath the cavity floor during the deep preparation in order to avoid pulpal exposure [65–67]. Iino et al. evaluated SS-OCT capacity for use in endodontic therapy. They found that SS-OCT could image the location of the second mesiobuccal canal in maxillary molars in vitro [68].

SS-OCT provides instant imaging with high resolution, which facilitates simultaneous chair-side diagnosis. One of the main advantages of OCT imaging over X-ray radiography is the ability to decrease the radiation dose from visual diagnostic approaches in dentistry. When there is doubt about the existence of a lesion, additional SS-OCT images with an altered position or angle can be obtained immediately. From this standpoint, SS-OCT is obviously the safer diagnostic modality that can be used for dental diagnosis on patients such as pregnant women and young children.

## Tooth Fracture Diagnosis

Tooth fractures have been a diagnostic challenge because of the difficulty in locating crack lines of incomplete tooth fractures [15, 16, 17••]. A tooth fracture can grow longitudinally with increased load to penetrate into the dentin and can lead to pulpitis and periodontal lesions as a result of bacterial leakage [15, 16]. Since a fracture has an unpredictable prognosis, including extraction, accurate diagnosis regarding the size and localization is required to determine the most appropriate treatment [15, 16, 17••].

Tooth fractures have been categorized into five major classes: craze line, fractured cusp, cracked tooth, split tooth, and vertical root fracture [69]. Craze line is the initial tooth crack, and asymptomatic itself, which occurs in the enamel surface parallel to the prismatic orientation by occlusal forces or thermocycling [69]. A cracked tooth is defined as a crack extending from the occlusal surface of the tooth apically without separation of the two segments [69].

Although many clinical diagnostic tools are available for tooth fractures, current methods such as radiography, transillumination, methylene blue dyes, and operative microscopic examination have limitations, and the uses of dental radiography or microtomography are controversial [15, 16, 70, 71]. Imai et al. employed SS-OCT for detecting naturally formed enamel cracks in vitro. The results for the detection of enamel cracks and whole-thickness enamel cracks in SS-OCT were calculated and compared with that of transillumination. SS-OCT showed superior results compared with transillumination for detection of enamel cracks and whole-thickness enamel cracks. Moreover, the reproducibility of SS-OCT among the three operators was significantly high [17••].

It is well known that the amount of reflection is dependent on the contrast of the refractive indices (η) of the media involved [51, 61, 72]. In a previous study of enamel and dentin measurements by OCT using an optical path-length-matching method, the η values were calculated as 1.63 and 1.55, respectively [61, 72]. For detection of tooth cracks, the crack space was assumed to be filled with dentinal fluid or water, for which η was approximately 1.3. Nonetheless, this discrepancy of η results was significant for signal peaks at the fracture border [17••, Fig. 2]. The enamel cracks on the SS-OCT images were clearly distinguished as a bright line because of the increased OCT signal intensity. Even when cracks extended beyond the DEJ, the whole line could be imaged through OCT, thus aiding determination of the crack penetration depth [17••].

Fried et al. showed that cracks can be visualized using near-infrared imaging at 1300 nm in in vitro and in vivo images [73]. Nevertheless, SS-OCT can construct a cross-sectional image of internal tooth structures within the range of the NIR penetration depth. In another study, it has been confirmed that SS-OCT could detect vertical root fractures of extracted human teeth [74]. However, the current SS-OCT setup has some limitations for detecting root fractures at the subgingival zone through an open root canal because of the probe design and through the gingiva because of signal attenuation in the soft tissue and bone [17••, Fig. 2]. Application of SS-OCT for tooth fracture detection is limited to the coronal portion in which laser light can be irradiated [17••, 73].

Shemesh et al. showed that OCT can be used to detect vertical root fractures using an inter-catheter rotating-pullback scanning probe [75]. Consequently, further development of technology, such as systems with improved transmission into soft tissue and special imaging probes for detection of the subgingival zone, can improve the diagnostic accuracy for detecting a tooth fracture and enhance the demands of OCT for clinical use.

## Detection of Gap Formation and Secondary Caries at the Restoration–Tooth Interface

Dentistry has been revolutionized by the introduction of resin-based materials that can bind to the tooth structure [76]. Although contemporary adhesives and composites provide excellent bonding to the tooth substrate, their major shortcoming is polymerization contraction that generates stress, leading to gap formation at the restoration–tooth interface [77]. Insufficient sealing may lead to leakage of oral fluids along the interface between the restorative material and tooth substrate and can result in postoperative tooth sensitivity, marginal discoloration, and recurrent caries [18, 76, 77]. OCT will also help assess the quality of restorative procedures and results during and after placement.

Dental X-rays are frequently used in the clinic to detect a translucent zone that can be associated with the presence of a thick adhesive layer, secondary caries, or a gap [78]. However, such loss of the interfacial seal appears undetectable by conventional dental X-rays because the size of these gaps has been reported to be ranging from 0.3 to 16 μm [79]. Makishi et al. evaluated marginal adaptation of composite restorations in class I cavities using SS-OCT and showed that three-dimensional imaging by SS-OCT can be considered a noninvasive technique for rapid detection of gaps at the restoration interface [19].

Cross-sectional SS-OCT images between the resin composite and dentin showed an increase in the signal intensity displayed as a clear line in the gray scale two-dimensional image, in which there was lack of an interfacial seal [18–20, Fig. 3].

Bakhsh et al. tried to quantify the interfacial gaps of the resin composite at the cavity floor of boxed shape class I cavities [20]. The SS-OCT images across the cavity floor were subjected to image analysis to determine the target pixels with significantly higher brightness. As a result, SS-OCT showed an acceptable sensitivity in gap detection at the walls [20, 80].

SS-OCT with an axial resolution of 11 μm would accurately measure gaps only a few micrometers in height because the Fresnel reflections at the gaps were detected even when the gaps were as small as half a micrometer in height, which is well below the SS-OCT axial optical resolution (11 μm) and vertical dimension of each image pixel (6.48 μm) [18, 20, 80]. Apart from the visualization of gaps at the interface, OCT imaging can also detect voids or air bubbles of different sizes within the composite restoration [81–83].

SS-OCT has been recently introduced to quantify interfacial gaps and evaluate the bonding interface [80, 84–87]. When the wall inclination was more than 35° to the incident laser beam, the dimension of the bright line at the cavity wall in the SS-OCT image correlated well with the actual gap size [85]. On the other hand, structures located parallel to the laser beam were not visible [85, 87].

## Conclusion

OCT is a promising imaging modality to noninvasively visualize dental caries, tooth fractures, and interfacial gaps in restorations without radiation. OCT can be a reliable and an accurate method and a safer alternative to X-ray radiography. Over the past decade, many functional OCT systems that provide specific optical characteristics have been reported for new biomedical research applications. In the near future, further technological development will allow utilization of OCT in soft tissue imaging for diagnosing periodontal disease and oral cancer.
